# Transient Increased Risk of Shingles Post–Shingrix Vaccination: Self-Controlled Case-Series Analysis

**DOI:** 10.1093/cid/ciaf473

**Published:** 2025-09-09

**Authors:** Aishwarya N Shetty, Daneeta Hennessy, Gonzalo Sepulveda Kattan, Samar Ojaimi, Hazel J Clothier, Jim P Buttery

**Affiliations:** Epidemiology Informatics, Centre for Health Analytics, Melbourne Children's Campus, Parkville, Victoria, Australia; SAEFVIC, Murdoch Children's Research Institute, Parkville, Victoria, Australia; Epidemiology Informatics, Centre for Health Analytics, Melbourne Children's Campus, Parkville, Victoria, Australia; SAEFVIC, Murdoch Children's Research Institute, Parkville, Victoria, Australia; Epidemiology Informatics, Centre for Health Analytics, Melbourne Children's Campus, Parkville, Victoria, Australia; SAEFVIC, Murdoch Children's Research Institute, Parkville, Victoria, Australia; Monash Pathology, Monash Health, Clayton, Victoria, Australia; Department of Medicine, School of Clinical Sciences, Monash University, Clayton, Victoria, Australia; Epidemiology Informatics, Centre for Health Analytics, Melbourne Children's Campus, Parkville, Victoria, Australia; SAEFVIC, Murdoch Children's Research Institute, Parkville, Victoria, Australia; Department of Pediatrics, University of Melbourne, Parkville, Victoria, Australia; Epidemiology Informatics, Centre for Health Analytics, Melbourne Children's Campus, Parkville, Victoria, Australia; SAEFVIC, Murdoch Children's Research Institute, Parkville, Victoria, Australia; Department of Pediatrics, University of Melbourne, Parkville, Victoria, Australia; Infectious Diseases Department, Royal Children's Hospital, Parkville, Victoria, Australia

**Keywords:** immunization, shingles, zoster reactivation, real-world evidence, vaccine safety

## Abstract

**Background:**

Following the introduction of a funded recombinant shingles vaccine (RZV; Shingrix, GlaxoSmithKline) vaccination program in adults aged ≥65 years in Australia, clinician reports of shingles presentations shortly after vaccination emerged. We investigated whether there was an increase in shingles risk immediately post–RZV vaccination in southeastern Australia.

**Methods:**

Two independent datasets—a general practice dataset and a statewide linked dataset—were analyzed separately using self-controlled case series (SCCS) analyses with 21 days postvaccination as the risk window. The observation period was 1 January 2023 to 30 April 2025. Adults ≥18 years were included, with analyses stratified by age (<65 and ≥65 years) and sex. We calculated the rate of incident shingles in time periods relative to vaccination, along with attributable risk and the risk of postherpetic neuralgia (PHN).

**Results:**

The primary care SCCS analysis found an 11-fold increase (relative incidence, 10.96; 95% CI, 10.34–11.62; *P* < .0001) in shingles presentations within 21 days post–dose 1 of RZV vaccination in adults ≥65 in the general practice dataset only. No increase was detected in younger adults. Following dose 2, the risk of shingles presentations was reduced in all age groups. Vaccine recipients had a 73% reduction in shingles following 2 doses. PHN risk was not increased.

**Conclusions:**

There is a transient increase in shingles presentations shortly after dose 1 of RZV vaccination in adults ≥65 years; however, these cases are likely to be mild and there is clear evidence of vaccine effectiveness after the completion of 2 doses.

Summary A transient increase in shingles presentations was observed following dose 1 of recombinant zoster vaccine in adults aged ≥65 years, with no increased risk of postherpetic neuralgia and strong evidence of vaccine effectiveness after 2 doses.

Herpes zoster, commonly referred to as shingles, is the reactivation of latent varicella zoster virus (VZV) in dorsal root ganglia, with migration down the nerve root, causing a painful vesicular rash in 1 or more dermatomes [1, 2]. In Australia, 2 herpes zoster vaccines are licensed: the live-attenuated zoster vaccine (ZVL; Zostavax; Merck & Co.; single-dose, now no longer available) and the adjuvanted recombinant inactivated subunit zoster vaccine (RZV; Shingrix; GlaxoSmithKline; 2 doses). Live-attenuated zoster vaccine was replaced by RZV on the National Immunization Program (NIP) in November 2023 [3]. Individuals aged 65 years and older were eligible for RZV under the funded NIP based solely on age-related risk and individuals aged 18 and older were eligible if they met specific clinical eligibility criteria—namely, they were considered at increased risk of herpes zoster due to an underlying condition and/or immunosuppressive treatments, as well as Aboriginal and Torres Strait Islanders aged 50 years and older. Self-funded vaccination outside these criteria was also possible [3]. Following the implementation of nationally funded RZV in adults aged 65 years and older in Australia, numerous anecdotal reports from physicians and individual cases of shingles onset proximal to vaccination have suggested that shingles vaccination has potential to cause VZV reactivation [4–6]. Disproportionality analyses of the Therapeutic Goods Administration also raised the possibility of a safety signal (Therapeutic Goods Administration, personal communication, 30 July 2024). To assess for evidence of an increased risk of shingles in the weeks immediately following RZV vaccination, we analyzed 2 independent data sources [7 , 8].

## METHODS

### Data Sources

#### General Practice Data

General practice (GP) data were routinely collected and curated via the Population Level Analysis and Reporting (POLAR) platform with permission and approval from participating Primary Health Networks in Victoria and New South Wales (NSW), Australia—namely, Central and Eastern Sydney, Eastern Melbourne, Western Victoria, Gippsland, Southeastern Melbourne, and Southwestern Sydney Primary Health Networks. The Primary Health Networks–owned deidentified dataset consists of 12 million electronic health records that are routinely collected [8, 9].

#### Linked Hospital and Laboratory Data

The Vaccine Safety Health Link (VSHL) is a repository of linked statewide administrative health datasets in Victoria, Australia, which has a population of 6.8 million [7]. The VSHL includes the Australian Immunisation Register records of Victorian residents, hospital records from the Victorian Emergency Minimum Dataset and the Victorian Admitted Episodes Dataset, notifiable condition and pathology records from the Public Health Event Surveillance System (PHESS), and death records from the Victorian Deaths Index [10]. Data linkage is conducted by the Centre for Victorian Data Linkage.

#### Identification of Shingles Diagnoses

In GP data, incident shingles cases were defined by Systematized Nomenclature of Medicine – Clinical Terms (SNOMED CT)-coded diagnoses of herpes zoster (including auricularis and ophthalmicus), with a subset also prescribed antivirals (acyclovir, famciclovir, or valaciclovir) within 7 days of shingles diagnosis. In hospital data, cases were identified by emergency department visits or admissions with International Statistical Classification of Diseases and Related Health Problems, Tenth Revision, Australian Modification (ICD-10-AM), codes starting with B02. In notifiable conditions data, it was defined as a confirmed or probable notification of shingles or varicella zoster infection—unspecified (PHESS). Repeat presentations within 180 days were excluded to capture incident cases.

#### Methodology

We applied 2 methodologies: (1) self-controlled case series (SCCS) analysis and (2) calculated rate of incident shingles in time periods relative to vaccination. Additionally, in the GP data we also calculated the attributable risk and the risk of postherpetic neuralgia (PHN) post-shingles. Furthermore, we calculated the rate of incident shingles in the unvaccinated population. The study period was 1 January 2023 to 30 April 2025 (Supplementary Figure 1). The observation period was censored at death in hospital/laboratory data, but not in GP data due to limited death reporting. To explore similar effects, we conducted a control analysis on ZVL recipients using the same methods from 1 January 2021 to 15 September 2024. All adults aged 18 years and older were included in the GP analysis and those aged 40 years and older included in the hospital and laboratory analysis. As laboratory tests cannot reliably distinguish chickenpox from shingles, excluding younger adults helped ensure results more likely reflected shingles.

**Figure 1.**
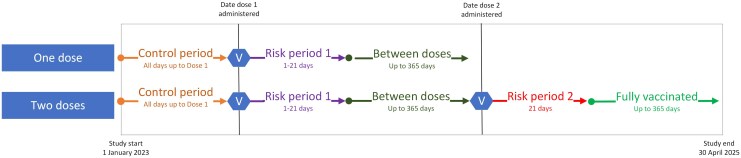
Risk and control windows in the SCCS analysis. Abbreviations: SCCS, self-controlled case series; V, vaccination day.

For the rates calculation, the population at risk comprised all people who had 1 or 2 doses of RZV. The day of vaccination was designated day 0 and the risk window following vaccination for each dose was day 1–21. In the SCCS, in our exposure–outcome context, the vaccine aims to prevent shingles, with protection expected shortly after the risk window. Including post-risk periods as control time could underestimate the relative incidence due to this protective effect. To minimize bias, we conservatively restricted control time to the prevaccination period. Follow-up was truncated at 365 days post–dose 1 or post–dose 2, depending on the number of doses received (Figure 1). We also conducted a sensitivity analysis excluding the 365 days before vaccination to reflect Australia's 12-month wait recommendation after a shingles diagnosis.

For the rates calculation, person-time at risk was calculated in periods relative to the date of vaccination, as follows:

1. Prevaccination period 1: start of study period—366 days before dose 1 administered
2. Prevaccination period 2: 365 prior-days of dose 1 administration
3. Risk period 1: day after dose 1 to 21 days after dose 1; if dose 2 was given earlier, the risk period was truncated on the day dose 2 was administered
4. Between-doses period: for people who received 2 doses, 22 days after dose 1 to the day of dose 2; for people who only received 1 dose, 22 days after dose 1 plus 365 days
5. Risk period 2: day after dose 2 administered plus 21 days
6. Postvaccination period: 22 days post–dose 2 plus 365 days

### Statistical Analysis

In the SCCS, the relative incidence (RI) of shingles was assessed for the 21-day risk window, the interval between doses, and the post–dose 2 period compared with a prevaccination control period. Analyses were conducted in R (version 4.2.3; R Foundation for Statistical Computing) using the SCCS and risks packages, with significance set at *P* < .01 [11–13].

For the rates calculation, the number of person-days at risk and the number of incident shingles events in the time periods were summed and converted to a rate of shingles per 1000 person-years. Events that occurred on the day of vaccination were not included in the primary analysis. However, a sensitivity analysis with inclusion of these events was performed (Supplementary Figures 2 and 3, Table 1). In the hospital and laboratory data, follow-up was censored at the date of death or 31 July 2024. Analyses were performed by age group (<65 years and ≥65 years) and sex.

We also calculated the attributable risk of shingles within 21 days post–RZV vaccination per 1000 first doses and assessed the overall change in shingles diagnoses before vaccination and after completion of the full vaccination schedule. To determine the risk of PHN post-shingles an analysis was performed for (1) shingles diagnosis but not RZV vaccinated, (2) shingles diagnosed within 21 days of RZV vaccination, and (3) shingles diagnosed more than 21 days after RZV vaccination or breakthrough case. A breakthrough case occurs when a fully vaccinated person still contracts the targeted disease. Postherpetic neuralgia is diagnosed when pain persists beyond 28 days of a shingles diagnosis [14]. We therefore excluded cases with PHN diagnosed within this 28-day window. The outputs were calculated as rate per 1000 for each cohort, risk estimates with 95% CIs, and stratified by age category [13]. Additionally, a sensitivity analysis including cases presenting with PHN diagnosed within 28 days of shingles was performed (Supplementary Figure 4).

### Ethics Approval

Ethics approval was granted by Monash Human Research Ethics Committee (HREC) (RES-18-0000-232A) for GP data, Royal Children’s Hospital Melbourne HREC (79964) for linked data, and Royal Australian College of General Practitioners – National Research Ethics and Evaluation Committee (RACGP NREEC) (17-008) for POLAR.

## RESULTS

The GP SCCS analysis included 7189 people with a new shingles diagnosis and at least 1 RZV vaccination; 93% (n = 6661) were 65 years or older (Supplementary Figure 5), 62% were female (n = 4498), and 60% (n = 4379) received 2 doses. Approximately 23% (n = 1687) were diagnosed with shingles within 21 days postvaccination, and 6% (n = 432) had multiple presentations over 180 days apart.

The hospital and laboratory SCCS included 3317 individuals with incident shingles and RZV vaccination; 64% received 2 doses (n = 2138), 85% were 65 years or older (n = 2846), and 61% (n = 2007) were female. Recurrence was rare (0.38%, n = 27). Among those aged 65 years and older, 53% of shingles events in GP data and 40% in linked data occurred within 1–7 days post–dose 1 (Supplementary Figure 6).

The SCCS analysis of the overall study population using GP data found an increased risk of shingles presentations in the 21 days following dose 1 RZV vaccination compared with the prevaccination period (RI, 10.96; 95% CI, 10.34–11.62; *P* < .0001). The hospital and laboratory data analysis found no significant difference (RI, 1.03; 95% CI, .85–1.31; *P* = .76).

In persons aged 65 years and older there was an increase in shingles presentations in the GP dataset (RI, 12.20; 95% CI, 11.49–12.94) post–dose 1. In the laboratory and hospital data the point estimate was over 1 but was not statistically significant (RI, 1.13; 95% CI, .92–1.39). Shingles risk was diminished in all other periods post–dose 2 (Table 1). There was no increase observed in either dataset in those aged younger than 65 years. No difference was found between sexes (Table 1).

**Table 1.** Relative Incidence of Shingles Post-Shingrix Compared With Prevaccination Period by Age Category and Sex in Southeastern Australia

	Including 365 Days Prior, RI (95% CI)					Excluding 365 Days Prior, RI (95% CI)				
	Overall	<65 y	≥65 y	Female	Male	Overall	<65 y	≥65 y	Female	Male
General practice (SNOMED CT only) (POLAR)										
Risk period 1	10.96 (10.34, 11.62)	.72 (.41, 1.26)	12.20 (11.49, 12.94)	10.60 (9.83, 11.42)	11.07 (10.06, 12.17)	7.01 (6.53, 7.53)	.48 (.27, .86)	7.73 (7.18, 8.31)	6.69 (6.11, 7.32)	7.24 (6.44, 8.13)
Between doses	1.41 (1.32, 1.51)	.81 (.61, 1.07)	1.47 (1.37, 1.57)	1.40 (1.29, 1.52)	1.43 (1.28, 1.60)	…	…	…	…	…
Risk period 2	1.58 (1.34, 1.86)	.15 (.03, .63)	1.76 (1.49, 2.07)	1.48 (1.20, 1.83)	1.68 (1.30, 2.17)	…	…	…	…	…
Fully vaccinated	.51 (.46, .56)	.11 (.06, .19)	.55 (.50, .61)	.54 (.48, .61)	.43 (.36, .51)	…	…	…	…	…
Linked hospital and laboratory dataset (VSHL)										
Risk period 1	1.03 (.85, 1.25)	.46 (.22, .97)	1.13 (.92, 1.39)	.98 (.75, 1.27)	1.17 (.87, 1.57)	.52 (.42, .64)	.27 (.13, .58)	.56 (.45, .69)	.49 (.37, .64)	.57 (.42, .77)
Between doses	.36 (.31, .42)	.34 (.22, .52)	.36 (.31, .43)	.36 (.29, .44)	.39 (.30, .49)	…	…	…	…	…
Risk period 2	.44 (.31, .62)	.18 (.04, .71)	.49 (.34, .70)	.54 (.36, .82)	.30 (.15, .61)	…	…	…	…	…
Fully vaccinated	.26 (.22,0.30)	.25 (.17, .37)	.26 (.21, .31)	.26 (.21, .32)	.28 (.21, .36)	…	…	…	…	…

POLAR GP data include Victoria and New South Wales data and VSHL linked data include only Victoria.

Abbreviations: GP, general practice; POLAR, Population Level Analysis and Reporting; RI, relative incidence; SNOMED CT, Systematized Nomenclature of Medicine – Clinical Terms; VSHL, Vaccine Safety Health Link.

Our sensitivity analysis excluding the 365 days prior to vaccination from the control window did not alter the findings in the GP dataset for all age groups. However, in the linked dataset, the point estimate was under 1 and significant (Table 1).

In the GP data, the subgroup of individuals with both a shingles diagnosis and an antiviral prescription represented 35% (n = 2528) of the total shingles cases. Among these, 88% (n = 2230) were aged over 65 years, 64% (n = 1620) were female, and 64% (n = 1618) had received their second dose of RZV. The SCCS analysis in those aged 65 years and older did not reveal an increased risk in the 21 days following vaccination (RI, 1.01; 95% CI, .80–1.26) (Table 2). There was no increased incidence of shingles in the 21 days following ZLV (<65 years: RI, .16; 95% CI, .02–1.23; ≥65 years: RI, .55; 95% CI, .38–.79; *P* < .01) (Supplementary Table 2).

**Table 2.** Relative Incidence of Shingles Post-Shingrix (With Antiviral Prescription) Compared With Prevaccination Period by Age Category and Sex in a General Practice Dataset Across Southeastern Australia

General Practice (SNOMED CT Only + Antiviral Prescription) (POLAR)	Including 365 Days Prior, RI (95% CI)					Excluding 365 Days Prior, RI (95% CI)				
	Overall	<65 y	≥65 y	Female	Male	Overall	<65 y	≥65 y	Female	Male
Risk period 1	1.01 (.80, 1.26)	.71 (.35, 1.46)	1.04 (.82, 1.32)	1.00 (.75, 1.33)	1.01 (.80, 1.26)	.52 (.41, .66)	.47 (.22, .98)	.53 (.41, .68)	.52 (.35, .78)	.52 (.39, .70)
Between doses	.44 (.37, .52)	.34 (.20, .56)	.45 (.38, .54)	.43 (.36, .53)	.44 (.37, .52)	…	…	…	…	…
Risk period 2	.39 (.25, .61)	.13 (.01, .96)	.43 (.28, .67)	.37 (.21, .65)	.39 (.25, .61)	…	…	…	…	…
Fully vaccinated	.37 (.31, .43)	.13 (.07, .25)	.41 (.34, .48)	.35 (.29, .43)	.37 (.31, .43)	…	…	…	…	…

POLAR GP data include Victoria and New South Wales data.

Abbreviations: GP, general practice; POLAR, Population Level Analysis and Reporting; RI, relative incidence; SNOMED CT, Systematized Nomenclature of Medicine – Clinical Terms.

In the GP dataset, 15% (n = 1067) received additional vaccines with RZV, with no shingles cases in the risk window. In the linked dataset, 8% (n = 250) had co-administration; 10 developed shingles in the risk window, representing 7% of all shingles diagnoses in the risk window. Common co-administered vaccines included influenza, coronavirus disease 2019 (COVID-19), and pneumococcal vaccines.

**Figure 2.**
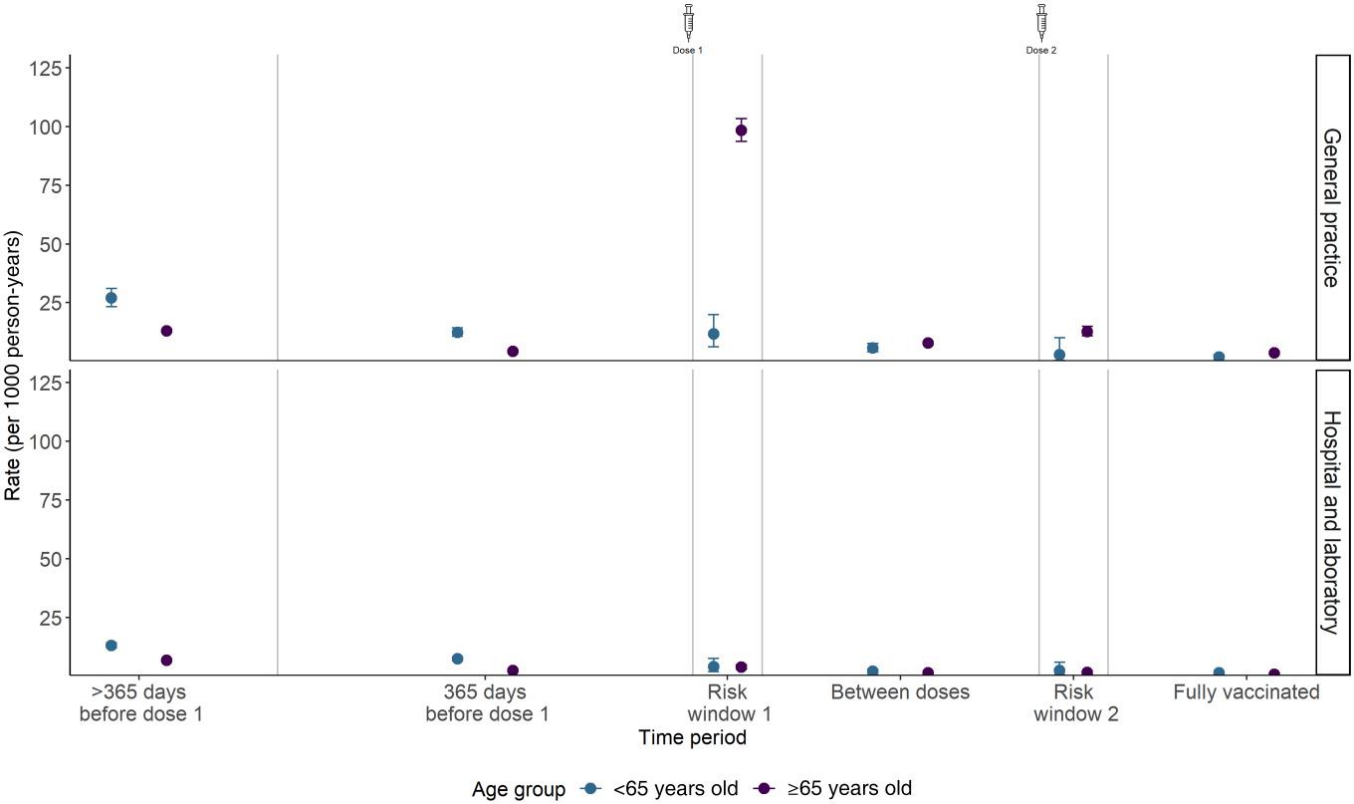
Rate of incident shingles in periods relative to vaccination.

For individuals aged 65 years and older in GP data, the rate of shingles in the 21 days following the first dose was higher compared with the periods before and after risk period 1. The shingles rate decreased relative to prevaccination after being fully vaccinated. This suggests that the risk is transient, brief, and likely mild and is elevated only in the short 21 days postvaccination (Figure 2). In the hospital and laboratory data, the rate during risk period 1 was higher than in the 1–365 days prevaccination period but lower than in the more-than-365 days prevaccination period, with a similar postvaccination decline observed (Figure 2).

The background rates in unvaccinated individuals aged younger than 65 years were similar in both datasets (GP, 2.0; hospital and laboratory data: 2.1 per 1000 person-years), whereas in the adults aged 65 years and older it was higher in the GP data (GP, 3.9; hospital and laboratory data: 2.8 per 1000 person-years).In adults aged 65 years and older, RZV was associated with 6.3 shingles cases per 1000 first doses in the 21-day risk window. In those younger than 65 years, rates decreased by 1.65 cases. Overall, there was a 73% (13.04 vs 3.59) reduction in shingles after completing the vaccination schedule (Figure 2).

**Figure 3.**
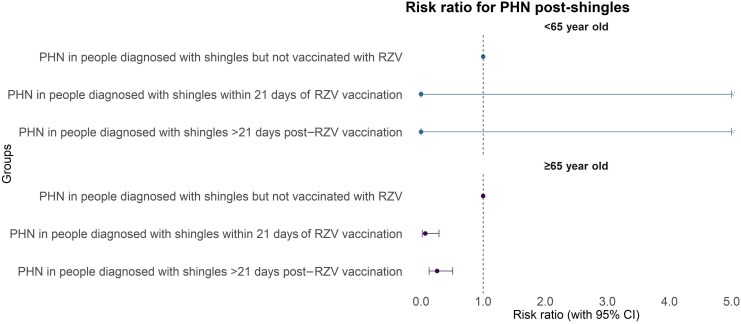
Risk estimates with 95% CIs by vaccination status and age category for PHN. Abbreviations: PHN, postherpetic neuralgia; RZV, recombinant shingles vaccine.

There was an approximate 75% reduction in PHN presentation in the persons who had shingles post–RZV vaccination compared with the unvaccinated population. No difference in PHN between those with shingles onset within 21 days compared with those with onset beyond 21 days was observed (Table 3, Figure 3). [2]

**Table 3.** Rate of Postherpetic Neuralgia Presentation per 1000 People Diagnosed With Shingles Post-RZV by Vaccination Status and Age Category

	<65 y		≥65 y	
	PHN Count/Total in Cohort	Rate per 1000 (95% CI)	Events	Rate per 1000 (95% CI)
PHN in people diagnosed with shingles but not vaccinated with RZV	116/23 676	4.89 (4.05, 5.87)	932/61 112	15.25 (14.29, 16.25)
PHN in people diagnosed with shingles within 21 d of RZV vaccination	0/15	0	2/1806	1.10 (.13, 3.99)
PHN in people diagnosed with shingles >21 d post–RZV vaccination	0/111	0	8/2048	3.90 (1.68, 7.68)

Abbreviations: PHN, postherpetic neuralgia; RZV, recombinant shingles vaccine.

## DISCUSSION

We describe an increased risk of shingles in the 21 days following initial RZV vaccination in adults aged 65 years and older using routinely collected GP data with no similar risk in younger vaccine recipients. Our findings also demonstrate that the risk of developing PHN is not increased.

Shingles incidence after dose 1 was over 11-fold higher in GP data than in hospital/laboratory data (RI, 10.96 vs 1.00). This likely reflects the clinical nature of shingles occurring after vaccination, which is mild and managed primarily in GP rather than in hospitals [15]. Existing evidence suggests that GPs show good diagnostic accuracy for herpes zoster [16]. Recent German data investigating zoster-like rashes presenting following RZV vaccination found 37.5% of eligible samples taken by GPs positive for varicella DNA. Even allowing for potential misclassification decreasing the magnitude of the signal, the increase observed in new diagnoses of shingles is likely to reflect a real increase in incident cases. These are likely to be mild in nature, given no observed increase in antiviral prescriptions or presentations to a hospital [17].

Linked data have low bias but likely underestimate shingles incidence, as most cases do not require hospital care or laboratory tests. General practice data capture more cases but are less specific. The true postvaccination increase likely falls between the estimates from both datasets.

In those individuals aged 65 years and older prescribed antivirals, the association was not significant. Shingles is usually diagnosed clinically in GP without swabbing [16] and antivirals are most effective when prescribed within 72 hours of symptom onset. Delays in seeking care, comorbidities, or clinical judgment may influence prescribing. Importantly, we found no increase in presentations post-Zostavax, suggesting no systematic overestimation of cases. Previous Australian data indicate that most community shingles cases are not treated with antivirals [18].

The exact cause of VZV reactivation after shingles vaccines is not fully understood [2] but may involve several factors. Vaccines can transiently weaken or alter cellular immunity, allowing latent VZV to reactivate [19, 20]. Immunosenescence, an age-related immune decline starting in an individual’s 60s, affects both innate and adaptive immunity [21]. The RZV vaccine's AS01B adjuvant strongly stimulates innate responses and suppresses natural killer (NK) cells within hours of vaccination [22]. We hypothesize that, especially in those with immunosenescence or compromised immunity, this immune activation may reduce VZV surveillance or redirect CD8 T cells to the vaccine, creating a window for reactivation [23].

One potential explanation for a higher risk post–dose 1 is that, by the time individuals receive dose 2, the immune system has already been primed by the first dose and may be better equipped to respond without triggering the same inflammatory pathways that could contribute to herpes zoster reactivation.

One reason we may not have observed an increased risk of zoster in the immunocompromised younger-than-65-years group is that, although this population has a heightened risk compared with healthy peers of the same age, their absolute risk may still be lower than that of older adults. Additionally, not all recipients younger than 65 years were actively immunocompromised at the time of vaccination.

Although shingles risk increases with age, higher rates in the younger-than-65-years group during the prevaccination period likely reflect the inclusion of clinically vulnerable individuals, such as those eligible for early RZV vaccination due to immunosuppression or other risk factors.

An analysis of GSK's global postlicensure data found that most adverse events after RZV were nonserious (95.3%), with shingles being the most common serious event (2.2 per 100 000 doses; 865 reports) [24]. A prior study of the same dataset, prompted by German postlicensure monitoring found shingles rates post-RZV lower than in the general population [20], although both relied on spontaneous reporting, limiting their validity [25].

Despite the brief 21-day period of increased risk, our results also demonstrate the subsequent effectiveness of RZV. Our GP findings show a brief period of increased shingles risk in the first 21 days after RZV dose 1, with an attributable risk of 6.3 cases per 1000 doses in adults over 65 years. However, when a person completes their vaccination schedule, there was a 73% reduction in the incidence of shingles within our study observation period (Supplementary Table 1). Additionally, the shingles rate among individuals who completed the vaccine schedule was similar to that in the unvaccinated population (3.59 vs 3.91 per 1000 person-years). Both data sources relied on clinical shingles diagnoses. A 2020 German study found that 50% of GP-suspected cases were polymerase chain reaction (PCR) confirmed [17], while earlier US studies reported higher positive predictive value (PPV) of 91% [16] and a case-control study found the PPV to be 86% [26], supporting reasonable diagnostic accuracy overall.

Similar to our findings, prelicensure clinical trial data from Zoster Efficacy Study in adults ≥50 years (ZOE-50) and Zoster Efficacy Study in adults ≥70 years (ZOE-70) found an 88.8% reduction in PHN [27]. Our real-world data underline the benefits of vaccination as a reduction in PHN has profound implications on quality of life [28].

A possible limitation of the GP data is that they only include doses administered at GPs using the POLAR system, leading to an underestimation of the total doses administered and the true relative incidence of events following vaccination. Another limitation in this study is that Australians are advised not to receive the RZV vaccine if they have had shingles in the previous 12 months. However, this recommendation is frequently not followed in practice. In our GP dataset, 39% of individuals received the vaccine despite having a shingles diagnosis 1 to 12 months earlier (Supplementary Figure 7). This study cannot assess overall protection from day 1 post–dose 1 due to limited follow-up. Longer-term effectiveness should be evaluated as more data become available.

Excluding the 365 days before vaccination helps address bias from the 12-month delay recommendation post-shingles. This sensitivity analysis had no impact on GP findings but altered estimates in hospital/laboratory data for those aged 65 and older (Table 1). However, this exclusion greatly shortens the control period and may exclude many individuals.

As shingles and shingles vaccination mainly occur in older adults, not accounting for death may inflate risk time. However, censoring at death in hospital/laboratory data changed rates by 1% or less, suggesting minimal impact on GP results. Recombinant shingles vaccine given as postexposure prophylaxis may raise short-term shingles risk, potentially biasing results. This seems unlikely as most of the vaccinated cohort were temporally associated with RZV being listed on the free NIP for adults aged 65 years and older.

### Conclusions

We demonstrate an increased risk of shingles presentations in the 3 weeks following the first dose of RZV in adults aged 65 years and older in an Australian GP dataset. These episodes are likely to be mild, with no increased rate of antiviral prescribing or hospital presentations. Our results align with concerns raised by physicians and demonstrate that the risk is transient.

## Supplementary Material

ciaf473_Supplementary_Data

## Supplementary Data

Supplementary materials are available at *Clinical Infectious Diseases* online. Consisting of data provided by the authors to benefit the reader, the posted materials are not copyedited and are the sole responsibility of the authors, so questions or comments should be addressed to the corresponding author.
