# Supplementary material for: Transient Increased Risk of Shingles Post–Shingrix Vaccination: Self-Controlled Case-Series Analysis
Source: Clin Infect Dis. 2025 Sep 9;81(5):e446–53. doi: 10.1093/cid/ciaf473 (PMC12728290; doi:10.1093/cid/ciaf473)
Supplement: ciaf473_Supplementary_Data [file ciaf473_supplementary_data.docx]

**Appendix Figure 1: Frequency of RZV vaccinations administered in POLAR registered in general practice data across Australia from 01 January 2023-30 April 2025**


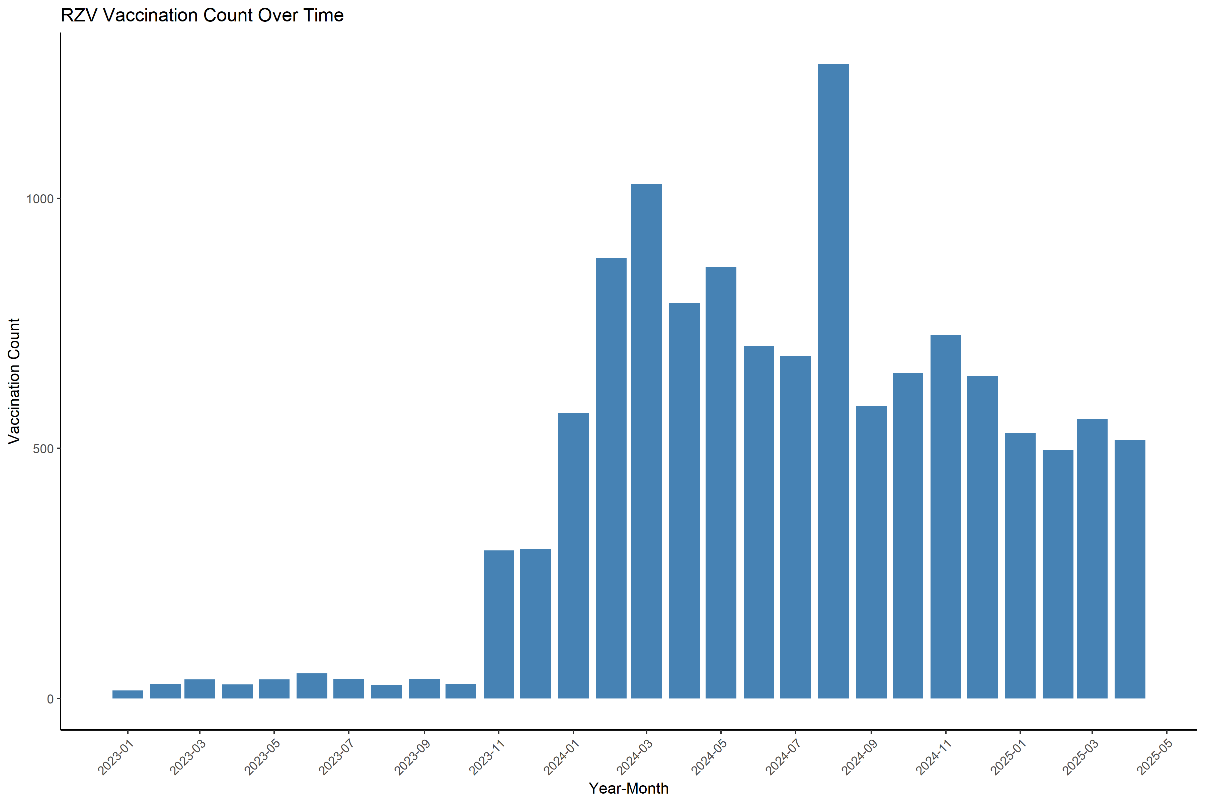


**Appendix Figure 2: Rate of incident shingles in periods relative to vaccination in general practice data (events on day zero counted in risk window)**


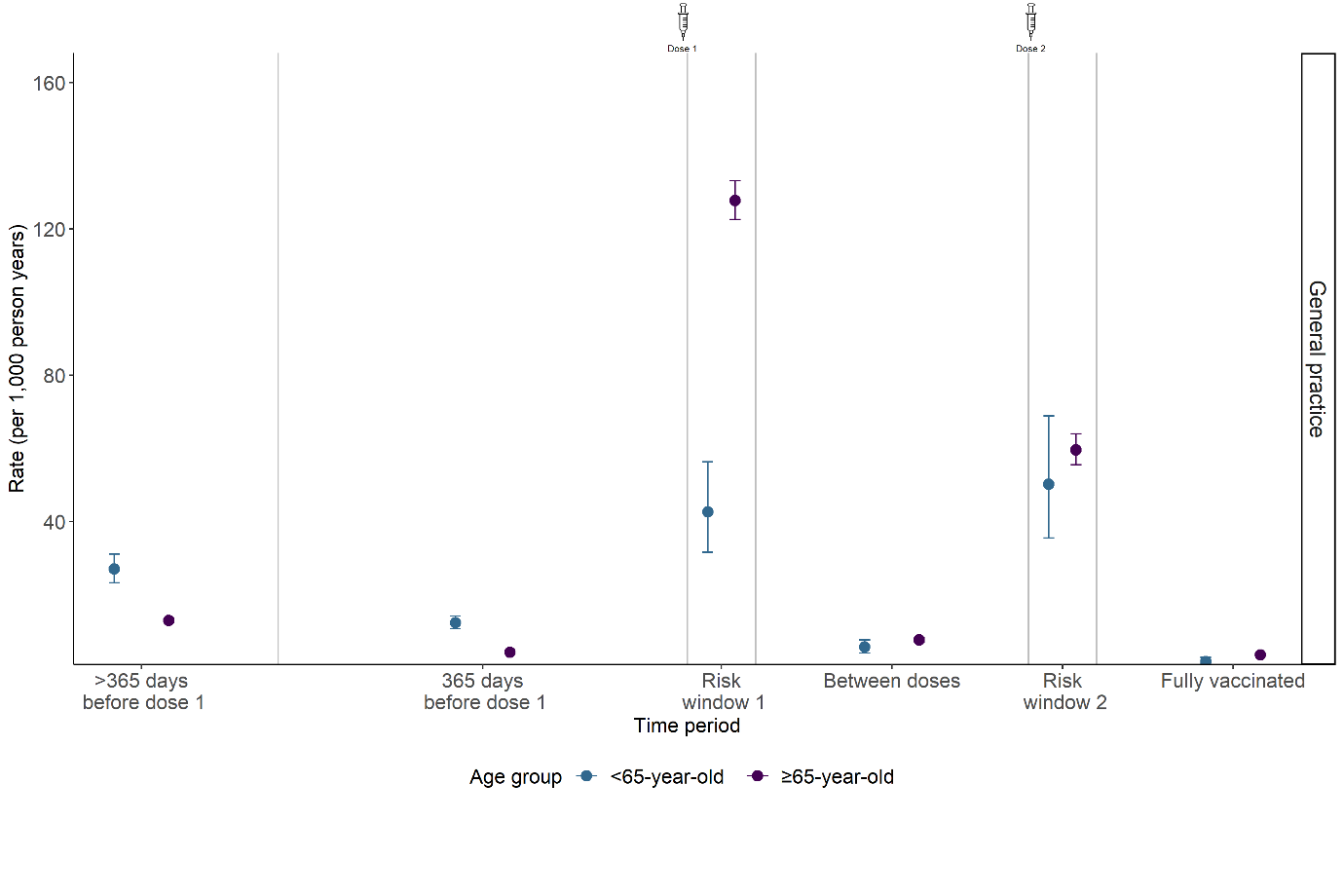


**Appendix Figure 3: Rate of incident shingles in periods relative to vaccination in general practice data (events on day zero counted in previous window)**


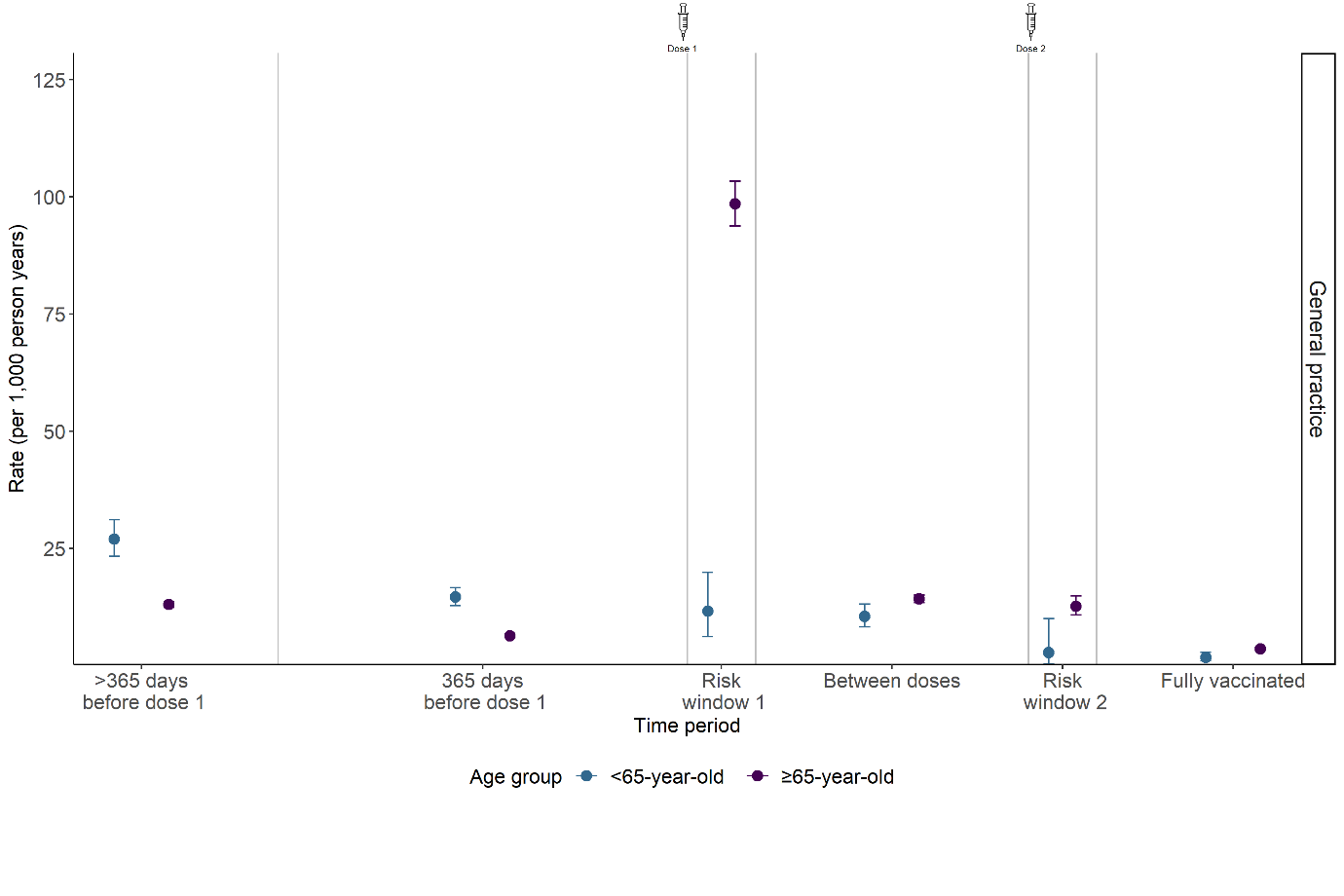


**Appendix Figure 4: Risk estimates with 95% confidence intervals by vaccination status and age category in general practice data (including diagnosis made within 28 days)**


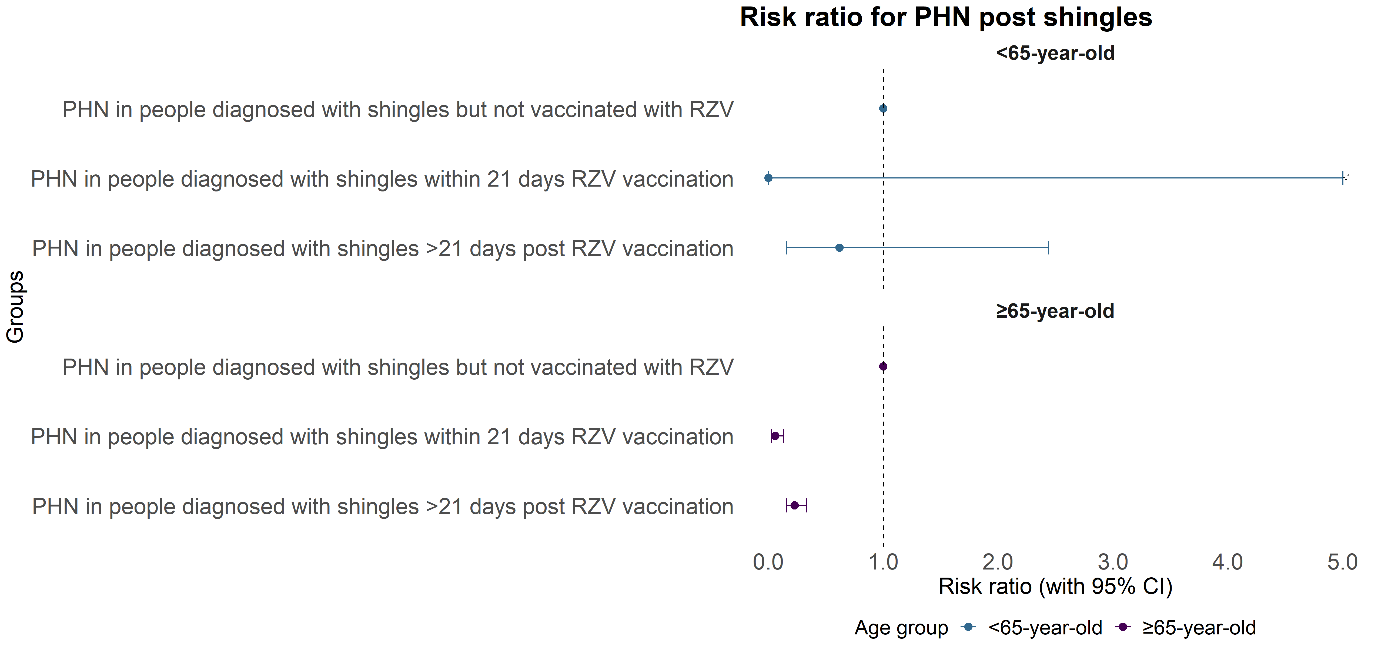


**Appendix figure 5: Age distribution in general practice and linked hospital and laboratory dataset**


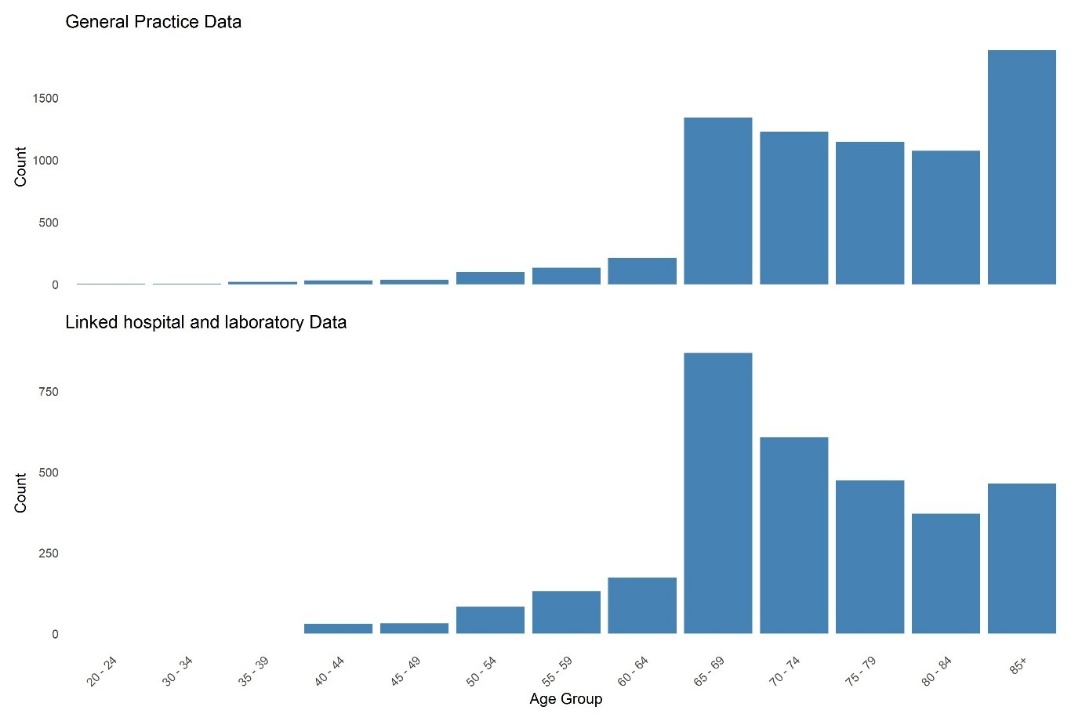


**Appendix Figure 6: Days between Shingrix vaccination and shingles diagnosis in general practice and linked hospital and laboratory dataset**


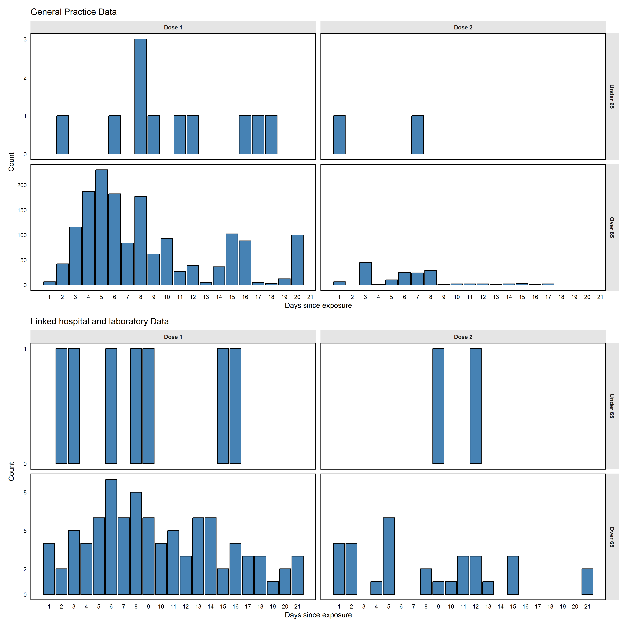


**Appendix Figure 7:** **Shingles diagnoses relative to Shingrix vaccination date in general practice and linked hospital and laboratory dataset**


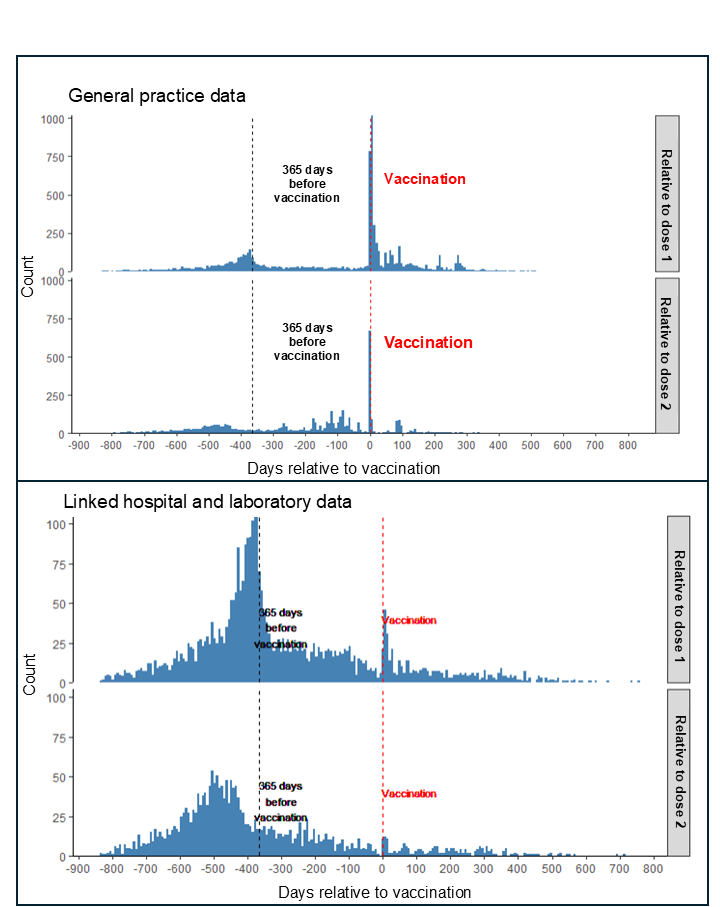


**Appendix Table 1: Counts for rate of incident shingles in periods relative to vaccination by age group**

| Source | Age group | Period | Events | Person time | Rate | 95% lower confidence interval | 95% upper confidence interval |
| --- | --- | --- | --- | --- | --- | --- | --- |
| Excluding events occurring on same day of vaccination | | | | | | | |
| Hospital and laboratory | ≥65-year-old | pre1 | 1438 | 166414 | 8.641 | 8.206 | 9.099 |
| Hospital and laboratory | ≥65-year-old | pre2 | 709 | 339330 | 2.089 | 1.941 | 2.249 |
| Hospital and laboratory | ≥65-year-old | risk1 | 74 | 18235 | 4.058 | 3.231 | 5.097 |
| Hospital and laboratory | ≥65-year-old | between | 119 | 90258 | 1.318 | 1.102 | 1.578 |
| Hospital and laboratory | ≥65-year-old | risk2 | 16 | 14434 | 1.109 | 0.679 | 1.809 |
| Hospital and laboratory | ≥65-year-old | post | 115 | 164648 | 0.698 | 0.582 | 0.839 |
| Hospital and laboratory | <65-year-old | pre1 | 262 | 15584 | 16.812 | 14.895 | 18.976 |
| Hospital and laboratory | <65-year-old | pre2 | 195 | 29773 | 6.550 | 5.692 | 7.537 |
| Hospital and laboratory | <65-year-old | risk1 | 7 | 1818 | 3.851 | 1.836 | 8.078 |
| Hospital and laboratory | <65-year-old | between | 25 | 10189 | 2.454 | 1.658 | 3.631 |
| Hospital and laboratory | <65-year-old | risk2 | 2 | 1502 | 1.331 | 0.333 | 5.323 |
| Hospital and laboratory | <65-year-old | post | 36 | 22231 | 1.619 | 1.168 | 2.245 |
| General practice | ≥65-year-old | pre1 | 1637 | 125584.8 | 13.04 | 12.41 | 13.68 |
| General practice | ≥65-year-old | pre2 | 1265 | 292808.3 | 4.32 | 4.09 | 4.57 |
| General practice | ≥65-year-old | risk1 | 1649 | 16753.12 | 98.43 | 93.74 | 103.3 |
| General practice | ≥65-year-old | between | 719 | 93121.86 | 7.72 | 7.17 | 8.31 |
| General practice | ≥65-year-old | risk2 | 157 | 12357.99 | 12.7 | 10.79 | 14.85 |
| General practice | ≥65-year-old | post | 526 | 146511.3 | 3.59 | 3.29 | 3.91 |
| General practice | <65-year-old | pre1 | 193 | 7143.06 | 27.02 | 23.34 | 31.11 |
| General practice | <65-year-old | pre2 | 198 | 15984.04 | 12.39 | 10.72 | 14.24 |
| General practice | <65-year-old | risk1 | 13 | 1114.49 | 11.66 | 6.21 | 19.95 |
| General practice | <65-year-old | between | 42 | 7370.22 | 5.7 | 4.11 | 7.7 |
| General practice | <65-year-old | risk2 | 2 | 720.72 | 2.78 | 0.34 | 10.02 |
| General practice | <65-year-old | post | 17 | 9537.21 | 1.78 | 1.04 | 2.85 |
| Events occurring on same day of vaccination included in risk window | | | | | | | |
| General practice | ≥65-year-old | pre1 | 1637 | 125,584.76 | 13.04 | 12.41 | 13.68 |
| General practice | ≥65-year-old | pre2 | 1265 | 292,808.34 | 4.32 | 4.09 | 4.57 |
| General practice | ≥65-year-old | risk1 | 2248 | 17,597.81 | 127.74 | 122.52 | 133.14 |
| General practice | ≥65-year-old | between | 719 | 93,121.86 | 7.72 | 7.17 | 8.31 |
| General practice | ≥65-year-old | risk2 | 774 | 12,980.46 | 59.63 | 55.5 | 63.98 |
| General practice | ≥65-year-old | post | 526 | 146,511.28 | 3.59 | 3.29 | 3.91 |
| General practice | <65-year-old | pre1 | 193 | 7,143.06 | 27.02 | 23.34 | 31.11 |
| General practice | <65-year-old | pre2 | 198 | 15,984.04 | 12.39 | 10.72 | 14.24 |
| General practice | <65-year-old | risk1 | 50 | 1,170.77 | 42.71 | 31.7 | 56.3 |
| General practice | <65-year-old | between | 42 | 7,370.22 | 5.7 | 4.11 | 7.7 |
| General practice | <65-year-old | risk2 | 38 | 757.09 | 50.19 | 35.52 | 68.89 |
| General practice | <65-year-old | post | 17 | 9,537.21 | 1.78 | 1.04 | 2.85 |
| Events occurring on same day of vaccination included in previous pre-vaccination period | | | | | | | |
| General practice | ≥65-year-old | pre1 | 1637 | 125584.8 | 13.04 | 12.41 | 13.68 |
| General practice | ≥65-year-old | pre2 | 1864 | 293653 | 6.35 | 6.06 | 6.64 |
| General practice | ≥65-year-old | risk1 | 1649 | 16753.12 | 98.43 | 93.74 | 103.3 |
| General practice | ≥65-year-old | between | 1336 | 93744.05 | 14.25 | 13.5 | 15.04 |
| General practice | ≥65-year-old | risk2 | 157 | 12357.99 | 12.7 | 10.79 | 14.85 |
| General practice | ≥65-year-old | post | 526 | 146511.3 | 3.59 | 3.29 | 3.91 |
| General practice | <65-year-old | pre1 | 193 | 7143.06 | 27.02 | 23.34 | 31.11 |
| General practice | <65-year-old | pre2 | 235 | 16040.32 | 14.65 | 12.84 | 16.65 |
| General practice | <65-year-old | risk1 | 13 | 1114.49 | 11.66 | 6.21 | 19.95 |
| General practice | <65-year-old | between | 78 | 7406.58 | 10.53 | 8.32 | 13.14 |
| General practice | <65-year-old | risk2 | 2 | 720.72 | 2.78 | 0.34 | 10.02 |
| General practice | <65-year-old | post | 17 | 9537.21 | 1.78 | 1.04 | 2.85 |

**Appendix Table 2: Relative incidence of shingles post Zostavax compared to pre-vaccination period by age category and sex in South-eastern Australia^#^**

|  | **Overall** | **<65-year-old** | **≥65-year-old** | **Female** | **Male** |
| --- | --- | --- | --- | --- | --- |
| Risk window | RI (95% CI) | RI (95% CI) | RI (95% CI) | RI (95% CI) | RI (95% CI) |
| Risk period | 0·51  (0·36, 0·73) | 0·16  (0·02, 1·23) | 0·55  (0·38, 0·79) | 0·59 (0·35,0·98) | 0·47  (0·29, 0·76) |
| Fully vaccinated | 0·23  (0·20, 0·27) | 0·06  (0·03, 0·15) | 0·25  (0·22, 0·29) | 0·24  (0·20, 0·30) | 0·23  (0·19, 0·28) |

^#^GP data include Victoria and NSW data and linked data includes only Victoria

**Appendix Table 3: Rate of post herpetic neuralgia presentation per 1,000 people diagnosed with shingles post RZV by vaccination status and age category (including diagnosis made within 28 days)**

| **Age categories(years)** | **<65-year-old** | | **≥65-year-old** | |
| --- | --- | --- | --- | --- |
|  | **PHN Count/Total in cohort** | **Rate per 1,000 (95% CI)** | **Events** | **Rate per 1,000 (95% CI)** |
| PHN in people diagnosed with shingles but not vaccinated with RZV | 692/23676 | 29.22  (27.11, 31.45) | 3,528/61,112 | 57.73  (55.89, 59.60) |
| PHN in people diagnosed with shingles within 21 days RZV vaccination | 0/15 | 0 | 6/1,806 | 3.32  (1.22, 7.21) |
| PHN in people diagnosed with shingles >21 days post RZV vaccination | 2/111 | 18.01  (2.18, 63.57) | 27/2,048 | 13.18  (8.70, 19.12) |
